# A complete workflow for single cell mtDNAseq in CHO cells, from cell culture to bioinformatic analysis

**DOI:** 10.3389/fbioe.2024.1304951

**Published:** 2024-02-19

**Authors:** Alan Foley, Nga Lao, Colin Clarke, Niall Barron

**Affiliations:** ^1^ Cell Engineering Group, National Institute for Bioprocessing Research and Training, Dublin, Ireland; ^2^ School of Chemical and Bioprocess Engineering, University College Dublin, Dublin, Ireland; ^3^ Bioinformatics Group, National Institute for Bioprocessing Research and Training, Dublin, Ireland

**Keywords:** Chinese hamster ovary, CHO, long-range PCR, single cell, mitochondrial DNA, mtDNA, single cell mitochondrial DNA sequencing, scmtDNAseq

## Abstract

Chinese hamster ovary (CHO) cells have a long history in the biopharmaceutical industry and currently produce the vast majority of recombinant therapeutic proteins. A key step in controlling the process and product consistency is the development of a producer cell line derived from a single cell clone. However, it is recognized that genetic and phenotypic heterogeneity between individual cells in a clonal CHO population tends to arise over time. Previous bulk analysis of CHO cell populations revealed considerable variation within the mtDNA sequence (heteroplasmy), which could have implications for the performance of the cell line. By analyzing the heteroplasmy of single cells within the same population, this heterogeneity can be characterized with greater resolution. Such analysis may identify heterogeneity in the mitochondrial genome, which impacts the overall phenotypic performance of a producer cell population, and potentially reveal routes for genetic engineering. A critical first step is the development of robust experimental and computational methods to enable single cell mtDNA sequencing (termed scmtDNAseq). Here, we present a protocol from cell culture to bioinformatic analysis and provide preliminary evidence of significant mtDNA heteroplasmy across a small panel of single CHO cells.

## 1 Introduction

**FIGURE 1:**
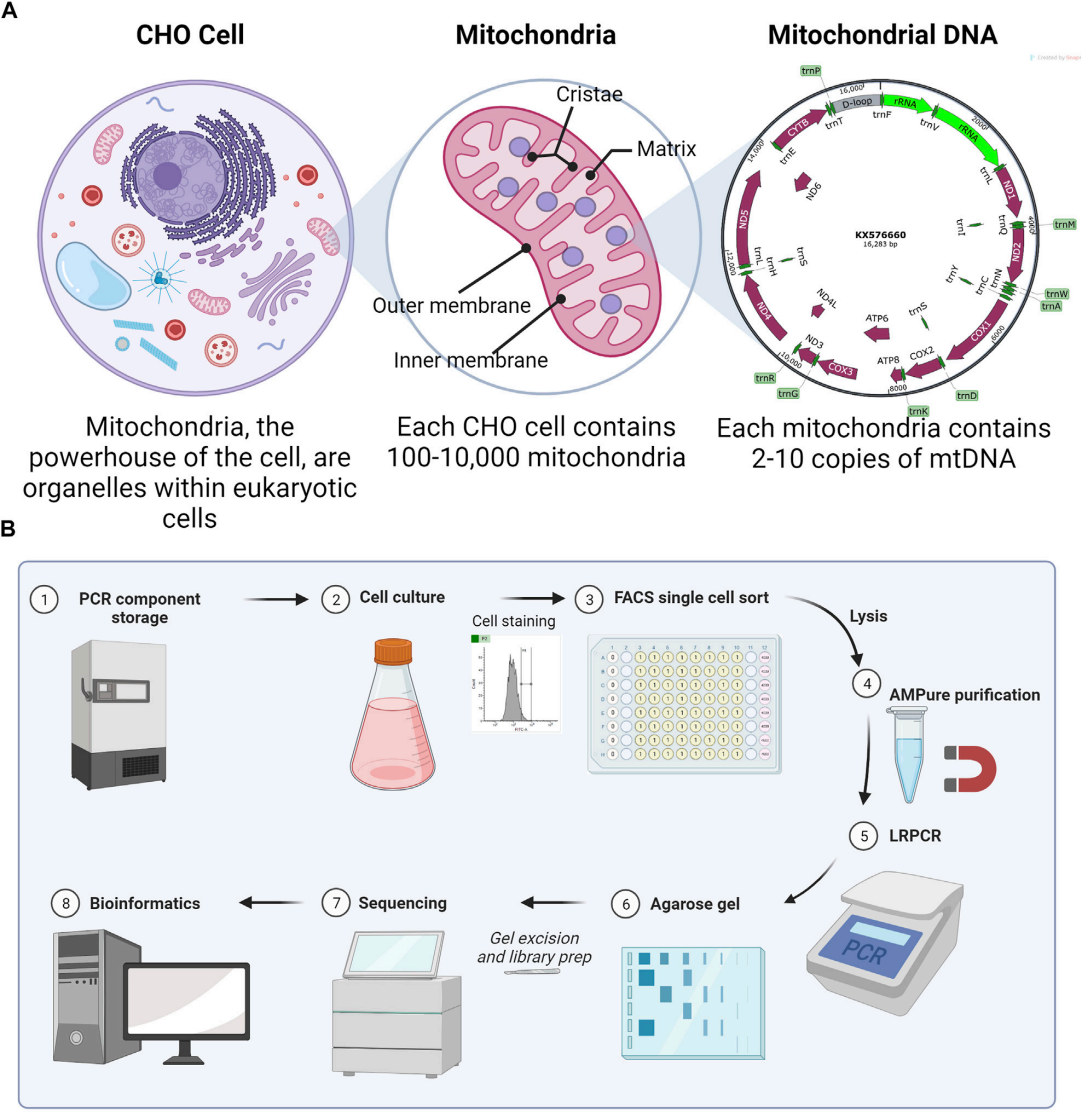
**(A)** Explanation of the multi-copy nature (heteroplasmy) of mitochondrial DNA. Numbers are true for CHO cells, although they vary with the eukaryotic cell type. **(B)** Method overview for scmtDNAseq. Made with BioRender.

Chinese hamster ovary (CHO) cells are the most commonly used mammalian host for the production of recombinant proteins (Walsh and Walsh, 2022). Optimization of biopharmaceutical production in CHO has led to titers routinely in the 3–8 g/L range (Kelley et al., 2018). Due to their importance in energy production, understanding the mitochondrial function in product-producing CHO cell lines is of particular importance. While most mitochondrial proteins are encoded by nuclear DNA, a small number of proteins are encoded by mitochondrial DNA (mtDNA). The CHO mitochondrial genome contains 37 genes, all of which support oxidative phosphorylation (OXPHOS). A total of 13 protein-encoding subunits are accompanied by 2 rRNAs and 22 tRNAs in a 16,283-bp plasmid-like circular structure (NCBI, 2023). mtDNA is highly compact, with the only significant non-coding region in the D-loop (Figure 1A).

Assuming a CHO cell has typical numbers of mitochondria per cell (100–10,000), each with 2–10 copies of mtDNA, the total genome copy per cell is large (Dhiman et al., 2019). In “homoplasmy,” all copies of mtDNA within a cell are identical; however, mitochondria can also exist in a state of “heteroplasmy,” where mutated versions of mtDNA co-exist with wild-type mtDNA within the same cell and possibly even within the same mitochondrion. *De novo* mtDNA mutations are common because of the 100x greater mutation rate than for nuclear DNA (Chial, 2008), explained by some due to the proximity to ROS (Kowaltowski and Vercesi, 1999) and by others due to mtDNA-replication errors (Itsara et al., 2014).

When the proportion of mutant mtDNA is above a particular threshold, mitochondrial dysfunction can occur (Dimauro and Davidzon, 2005). The phenotypic effect of heteroplasmic mutations increases as the proportion of mutant mtDNA (allele frequency) increases. Mouse models with an increasing allele frequency of a mutation in *ND5* showed increasing dysfunction in respiration, OXPHOS, and, ultimately, ATP generation (Park et al., 2009). A causal relationship has since been attributed to a plethora of mtDNA mutations in a wide range of diseases, particularly in cancer (Hertweck and Dasgupta, 2017), where mtDNA mutations are observed in 50% of tumors (Ju et al., 2014).

A previous bulk analysis identified heteroplasmy between clonal CHO cell populations (Kelly et al., 2017), laying a theoretical explanation for the metabolic heterogeneity often observed in CHO cell cultures (Gilbert et al., 2013). Single cell sequencing of mtDNA (scmtDNAseq) has previously been carried out in non-CHO cell lines using high PCR cycle numbers of 40 (Zambelli et al., 2017) and 45 (Maeda et al., 2020). Higher PCR cycle numbers are associated with a greater risk of undesirable secondary products, such as PCR artifacts (Lorenz, 2012). In a single cell mtDNA analysis, the starting mtDNA copy number is low (<100,000); therefore, even small contaminations can confound the accurate calling of mutations. To call heteroplasmic mutations at a 0.015 allele frequency (a conservative level), PCR amplification should ideally not exceed 30 cycles (Zambelli et al., 2017). Furthermore, Maeda et al. focused on specific mutations, not the whole mtDNA genome, precluding the identification of as yet unknown mutations. There is a real value in novel whole mtDNA single cell analysis with a low PCR cycle number.

Here, we sought to develop an optimized method to amplify the mtDNA and sequence from single CHO cells. To demonstrate the method, we analyzed four single CHO cells and a bulk (multiple cells) sample for comparison. Single cells were isolated by fluorescence-activated cell sorting (FACS) into lysis buffer with an emphasis on simple and reproducible gating (Figure 1B). After optimization of the lysis buffer, PCR kit, and purification system, the cycle number for long-range PCR (LRPCR) was kept lower (35x) than that of previously reported methods. Importantly, this provides more confidence in low-frequency heteroplasmy. To ensure the exclusion of contaminating nuclear mitochondrial DNA (Numts), primers were designed to exclusively map to CHO mtDNA and amplicons size-selected via gel electrophoresis. By confirming mtDNA amplification by agarose gel, we were able to improve the efficiency of our sequencing since only successful reactions were brought forward for library preparation. Illumina DNA libraries were generated, and iSeq 100-derived sequencing output was processed and analyzed using a bespoke bioinformatics pipeline. Preprocessing was performed in Linux and data analysis in R.

## 2 Materials and equipment

### 2.1 CHO cell culture

1. 125 mL bioreactor flasks (Nalgene, 10266432).
2. Appropriate CHO cell culture medium (e.g., Gibco CD FortiCHO, 10887640).
3. CHO cell lines of interest (e.g., Table 1).

### 2.2 Immunolabeling and staining

1. DPBS.
2. Nuclease-free water.
3. Trypan Blue 0.4% (Gibco, 15250061).
4. Luna II.
5. DAPI (Invitrogen, D1306).
6. Goat F(ab’)2-fluorescein anti-human IgG (Sigma Aldrich, SAB3701254-2MG) to label IgG-producing cells if desired. Other appropriate fluorescent stains could also be used.

### 2.3 FACS

1. 70% IPA.
2. FACS with appropriate lasers for DAPI and FITC detection. Here, the BD FACSMelody was used.
3. FACS polystyrene tubes (Falcon Corning, 1018640)
4. U-bottom 96-well plates (Corning, 3799)
5. Parafilm.
6. TCL buffer (QIAGEN, 1070498).

### 2.4 AMPure purification

1. AMPure XP beads (10136224).
2. 70% ethanol.
3. Elution buffer (QIAGEN, 19086).
4. Sterile PCR tubes (autoclaved).
5. 0.2 mL tube magnetic stand (New England Biolabs, S1515S)
6. 10-µL multichannel pipette (optional).

### 2.5 Long-range PCR

Primers were designed using NCBI Primer-BLAST to specifically bind to mtDNA and not to any known CHO nuclear DNA sequences to minimize Numt contamination.

1. SuperFi II Plat Taq (Invitrogen, 12361010).
2. PCR thermocycler.
3. 10-µM forward and reverse primers (Table 2) (IDT).
4. 10-mM dNTP Mix (Thermo Scientific, R0192).

### 2.6 Agarose gel

1. Agarose powder.
2. TAE buffer.
3. SafeView (NBS Biologicals). Ethidium bromide is an alternative.
4. GeneRuler 1-kb Plus DNA Ladder (Thermo Scientific, SM1333).
5. Gel Viewer/transilluminator.
6. Disposable laboratory scalpel.
7. Eppendorf tubes.

### 2.7 Gel purification

1. QIAquick Gel Extraction Kit (QIAGEN, 28706X4). Other gel extraction kits could also be utilized.

### 2.8 Qubit

1. Qubit 4 Fluorometer (Invitrogen).
2. Qubit 1x dsDNA HS Kit (Invitrogen, Q33230).

### 2.9 Sequencing

1. iSeq 100 (Illumina) PE150, 8 million reads.
2. Illumina DNA Prep, (M) Tagmentation (24 samples) (Illumina, 20018704).
3. IDT for Illumina DNA/RNA UD Indexes Set A, Tagmentation (96 indexes and 96 samples) (Illumina, 20027213).
4. iSeq 100 i1 Reagent v2 (300-cycle) (Illumina, 20031371).
5. PhiX v3 (Illumina FC-110-3001).

## 3 Methods

All steps up to the completion of the LRPCR for the four samples (Table 1) were performed in sterile conditions (BSC). CHO-K1 immortalised cell line from the European Collection of Authenticated Cell Cultures (ECACC)—(Cat#85051005).

### 3.1 PCR component storage

Since the LRPCR amplifies from <5,000 copies of mtDNA, PCR components must have an optimal efficacy. This was ensured by making small (20 µL) aliquots of dNTPs (Thermo Scientific, R0192) and primers (IDT) and storing them at −80°C. Fresh aliquots were used for each batch of PCR performed and subsequently discarded.

### 3.2 CHO cell culture

CHO-GS cells were cultured in FortiCHO (Gibco CD FortiCHO, 10887640) at 37°C, 5% CO_2_, 85% humidity, 1 and 25 rpm with 25-mm orbit in a shaking incubator in 125 mL bioreactor flasks (Nalgene, 10266432). Every 3–4 days, the cells were passaged at 0.2*10^6 cells/mL in 30 mL media in 125 mL culture shaking flasks. Viability was determined by Trypan Blue exclusion using a hemacytometer (Luna II). A growth curve was established to ensure samples were taken at the exponential cell phase (Table 1).

**TABLE 1:** Samples 1–4 required for single cell sorting. In these data, late exponential had a viability of 95% and dead of 5%.

	Cells	Growth phase	Stain	Function
1	Protein-producing CHO	Late exponential	DAPI + FITC-AB	Sorting sample
2	Protein-producing CHO	Dead	DAPI + FITC-AB	Gate live/dead cells
3	Non-producing CHO	Late exponential	DAPI + FITC-AB	Gate FITC-negative
4	CHO	Late exponential	None	Gate FSC and SSC; Gate FITC-positive

### 3.3 DAPI stain

A working concentration of 0.1 μg/mL DAPI was determined as optimal for CHO cells. DAPI (Invitrogen, D1306) solutions were protected from light wherever possible. In a BSC, 10 mg DAPI powder was completely dissolved in 2 mL sterile deionized water to make 5 mg/mL DAPI stock solution. This was aliquoted and stored at −20°C. The solutions have a stability period of at least 6 months. A measure of 1 µL of the DAPI stock solution was added to 5 mL DPBS to prepare 1 μg/mL stock 2 DAPI working solution. A measure of 1mL of 1 μg/mL stock 2 solution was added to 9 mL of DPBS to prepare 0.1 μg/mL DAPI working solution.

### 3.4 Staining cells

Here, an AB-FITC (Sigma Aldrich, SAB3701254-2MG) conjugate was used, which, at 4°C, can bind to IgG on the cell membrane in the process of being excreted by the cell, as previously demonstrated for CHO cells (Gallagher and Kelly, 2017). This allowed the sorting of cells based on the productivity of an IgG-based antibody. Cell samples were prepared according to Table 1. Cells were counted using Trypan Blue exclusion (Gibco, 15250061) and a hemacytometer (Luna II), as per the manufacturer’s instructions. Then, 1*10^6 of viable cells were centrifuged at 200 ×g for 5 min, and the supernatant was discarded. The cells were washed in 1 mL of DPBS and centrifuged at 200 ×g for 5 min, and the supernatant was discarded. This was repeated for a total of two washes. Cells were resuspended in 1 mL of DPBS using 2 µL of the AB-FITC. The cells were incubated at 4°C for 30 min, protected from light. The cells were washed twice with DPBS as per steps 6 and 7 for a total of two washes. The cells were resuspended in 1 mL of cold DPBS or cold DAPI working solution, incubated on ice for 5 min, and immediately transferred on ice to the fluorescence-activated cell sorting (FACS) laboratory for immediate analysis.

### 3.5 Setting single cell gating

**FIGURE 2:**
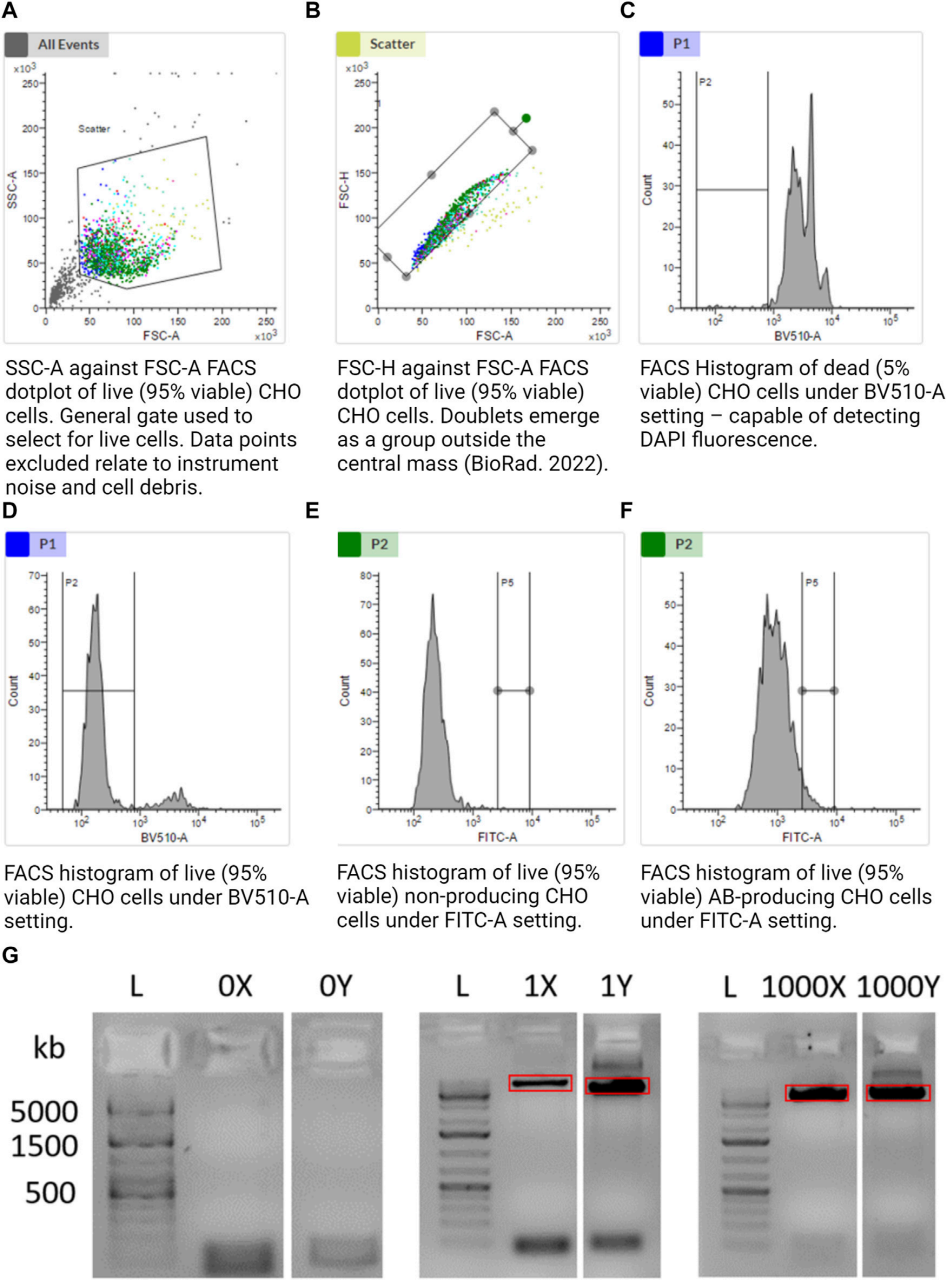
**(A–F)** Gating strategy to sort alive, singlet, and antibody-producing CHO cells. **(G)** Agarose gel illustrating the amplification of CHO cell mtDNA from a single cell. Further included are the positive control of 1,000 cells and negative control of 0 cells. “X” and “Y” refer to the two separate halves of the mtDNA molecule. Together, 1X and 1Y represent amplification of the whole mtDNA molecule from a single cell in two separate reactions. The red rectangles illustrate gel extraction boundaries to exclude bands other than the desired 8.5-kb amplicon. Made with BioRender.

FACS-based sorting was implemented as it allows cells to be chosen or discarded based on pre-defined traits, e.g., viability. FACSMelody was set up as per the manufacturer’s instructions. A U-bottom 96-well plate was prepared (Corning, 3799) with 5 µL of 1x TCL buffer (QIAGEN, 1070498) in the center of each functional well using a multichannel pipette. The plate was tapped firmly on a flat surface to encourage the central location of the TCL buffer. The size threshold was set to >12 µm. Using sample 4 (Table 1), voltages were set to allow the representation of cells in an SSC-A against the FSC-A logarithmic scale graph. Gate 1 (G1) excluded instrument noise and cell debris as per Figure 2A. Using sample 4, G1 data were selected, and gate 2 (G2) was set using FSC-H against FSC-A as per Figure 2B to exclude doublets. Using samples 1 and 2, gate G2 was selected, and a range gate (G3) was set to only include live cells as per Figures 2C, D. DAPI-positive was considered dead cells. Using samples 1 and 3, gate G3 was selected, and a gate (G4) was set for FITC-positive cells as per Figures 2E, F. G4 was the sorting gate for live, singlet cells. After the gates had been set, data were recorded for 10,000 cells to enable a good representation of the population. Cells that fulfilled our gating strategy were sorted into wells of a 96-well plate with lysis buffer.

We chose single cells with a high AB-FITC signal as these are the most relevant in a biomanufacturing setting, i.e., cells with a high specific productivity of the therapeutic antibody that they are synthesizing and secreting.

### 3.6 Single cell sorting

The flow rate was kept low (<1,000 events/second) to reduce the chance of doublets. Sample 1 was loaded with a splash shield present, and the FACS was set to “single cell” and “96-well plate” modes. Desired wells were selected for sorting. The lid was removed and immediately inserted into the FACS to proceed with sorting. For the positive control, the sort mode was changed to “purity.” After the sorting was complete, the well plate was removed and immediately covered with the lid. An airtight seal was created around the edges with parafilm, and the plate was immediately placed in a −80°C freezer for storage and to encourage further lysis. FCS files were saved for all samples.

Stopping point: Samples can be stored for up to 6 months at −80°C.

### 3.7 AMPure purification

In a previous bulk analysis of mtDNA, the miniprep step purified the plasmid-like mtDNA from contaminating linear nuclear DNA (Kelly et al., 2017). The miniprep kit concomitantly provided some protection against Numts since it is designed to purify circular mtDNA away from linear nuclear DNA (Kelly et al., 2017). Having eliminated the miniprep step, we sought to incorporate additional protection against Numts. We performed a BLAST search of our mtDNA amplification primer sequences against the nuclear CHO reference genomes and found no matches, suggesting that there are no nuclear sequences to which our primers should bind. Furthermore, Numts tend to be shorter sequences, with 78% shorter than 500 bp in human mtDNA (Wei et al., 2022). Therefore, we reasoned that specific gel purification of 8.5 kb amplicons would be unlikely to be contaminated with Numts.

All steps were performed in a BSC. The subsequent LRPCR is extremely sensitive and could potentially amplify small contaminations. The 96-well plate was thawed at room temperature. The 5-µL lysed sample was transferred to a labeled microcentrifuge tube. AMPure beads were resuspended by vortexing the bottle for 1 min. A measure of 9 µL of AMPure beads was added per sample (if the lysed cell sample was greater, 1.8× volume of AMPure beads was used) and pipette-mixed 10 times. They were left at room temperature for 5 min. Tubes were placed on a magnetic stand (New England Biolabs, S1515S) for 2 min. Keeping the tubes on the magnetic stand, the cleared solution was removed and discarded, leaving the beads. It was then washed with 40 µL 70% ethanol. The supernatant was discarded, leaving the beads. The ethanol wash was repeated. On the second wash, the remaining ethanol was removed using a P10 pipette while carefully avoiding the beads. The tubes were removed from the magnetic stand, and 18 µL of the elution buffer (QIAGEN 19086) was added to the bead aggregate and pipette-mixed 10 times or until fully resuspended. The tubes were incubated for 5 min at room temperature and then placed on the magnetic stand for 2 min. Eluate was split into two 8.5 µL aliquots, leaving the bead aggregate.

### 3.8 SuperFi II Plat Taq LRPCR

The bottleneck of our scmtDNAseq method was the DNA amplification step. Amplification techniques that would work for bulk sequencing proved to be incompatible with single cells: mechanical purifications took too much of the sample, bacterial lysis buffers did not release enough mtDNA, and components lost effectiveness for the sensitive PCR. However, we reasoned that once enough mtDNA was amplified, established protocols for bulk sequencing could then be followed (Kelly et al., 2017). The SuperFi II PCR kit (Invitrogen, 12361010) has ×300 fidelity compared to Plat Taq, which we reasoned would give us greater confidence in lower-level heteroplasmy. Higher fidelity also means greater confidence in lower-level heteroplasmy.

In addition to the below LRPCR protocol, single cell samples post-AMPure purification were diluted to 1/10, 1/100, 1/1,000, and 1/10,000 to demonstrate the limits of the high-fidelity LRPCR kit. All steps were performed in a BSC while maintaining the samples at all steps on ice. Fresh aliquots of primers and dNTPs were thawed at room temperature and then stored on ice. The SuperFi II 5x Buffer was thawed and stored on ice. DNA polymerase was maintained at −20°C and only removed briefly when needed. The components were briefly vortexed and centrifuged before use, except for the DNA polymerase. mtDNA LRPCR was performed in two separate fragments (termed X and Y). The eluate from a single cell had been split into two from AMPure purification; one half was amplified using X primers, and the other half, by Y primers (Table 2).

**TABLE 2:** Primer sequences for LRPCR. Other cell lines may need adaptation of these sequences.

Primer	Sequence
mt-490 F (X)	5’–GGA TTA GAT ACC CCA CTA TGC TT–3′
mt-9304 R (X)	5’–ATG CTG CGG CTT CAA ATC CG–3′
mt-9180 F (Y)	5’–ATA GCA ACA GGT TTT CAC GG–3′
mt-598 R (Y)	5′–CGC CAA GTC CTT TGA GTT TTA–3′

The primers were designed using NCBI Primer-BLAST to ensure specificity for mtDNA and no targeting of known nuclear DNA regions in CHO reference genomes. A mastermix was generated with 10% overage for each X primer and Y primer, as per the example in Table 3. SuperFi II DNA polymerase was added last by briefly removing it from the −20°C freezer to minimize the time spent at room temperature. The mastermix was gently vortexed, centrifuged at 500 ×g, and kept on ice. A measure of 16.5 µL of the mastermix was added to 8.5 µL of AMPure purified DNA. The sample was gently vortexed, centrifuged at 500 ×g, and maintained on ice. The samples were placed in a PCR machine and set to a PCR cycle, as per Table 4. The reaction volume was set to 25 µL with a lid temperature of 105°C. The cycle was run overnight. On completion, the samples were removed and stored at 4°C.

**TABLE 3:** PCR components.

Reagent	Volume per rx (µL)	10x Mastermix (µL) X	10x Mastermix (µL) Y
5x Buffer	5	50	50
10 mM DNTP mix	0.5	5	5
10 µM Primer F	1	10 (X primer)	10 (Y primer)
10 µM Primer R	1	10 (X primer)	10 (Y primer)
Nuclease-free H_2_O	8.5	85	85
SuperFi II DNA polymerase	0.5	5	5
TOTAL	16.5	165	165

**TABLE 4:** PCR settings.

Step	Temperature (°C)	Time
1. Initial denature	94	2 min
2. (x35) Denature	94	30 s
Annealing	55	30 s
Extension	68	9 min
3. Final extension	68	10 min
4. Hold	4	Infinite hold

Stopping point: Samples can be stored at −20°C for 2 weeks.

### 3.9 Agarose gel

An amount of 1 g of agarose was added to 100 mL TAE buffer in a conical flask and microwaved for 2.5 min or until fully dissolved. The flask was left to cool to approximately 50°C. A measure of 10 µL of SafeView (NBS Biologicals) was added, and the mixture was poured into a gel tray with a well comb. After a brief period, the gel cooled and hardened at room temperature. The gel was placed in a gel box with TAE buffer just covering the gel. The loading dye was added to all samples as per the manufacturer’s instructions. Entire samples were loaded into gel wells with an appropriate DNA ladder (Thermo Scientific, SM1333). The gels were run at 100 V until the bands were 70% down the gel. The power was turned off, and the gel was carefully placed in a gel viewer. Photographs of the gel were taken.

Limit of detection: an 8.5-kb band was still observable when taking a 1/1,000 dilution of a single cell (Supplementary Figure S1A). We would expect around 100–10,000 mitochondria per cell (Dhiman et al., 2019), implying that this method may even be viable for single cell mitochondrial sequencing.

### 3.10 Gel excision

Under a gel visualizer, 8.5-kb bands were observed, indicative of single cell reactions, as illustrated by red rectangles in Figure 2G. UV light exposure was minimized to limit DNA degradation. Using a new sterile disposable scalpel, the 8.5-kb band was excised and placed in a 1.5 mL Eppendorf tube. The blade was thoroughly cleaned with 70% IPA and then reused. The Y single cell sample was equally isolated and placed in a separate 1.5 mL Eppendorf tube.

### 3.11 Gel purification

The “QIAquick gel extraction using a microcentrifuge” protocol was used to purify from agarose gels. Only 10 µL of elution buffer was used to encourage a higher final concentration.

Stopping point: Samples can be stored at −20°C for 2 weeks.

### 3.12 Equimolar combination

The Qubit 1x dsDNA HS kit (Invitrogen, Q33230) was used to quantify dsDNA. Kit components were allowed to equilibrate to room temperature for 30 min. A measure of 10 µL of standard 1 was added to a Qubit tube, and 10 µL of standard 2, to a separate Qubit tube. Then, 190 µL of 1x buffer was added to each. Thereafter, 1 µL of each X and Y fragment was added to the separate Qubit tubes, and 199 µL of 1x buffer was added to each. The tubes were vortexed for 2–3 s and left at room temperature for 2 min. The concentration of standards 1 and 2 were measured using the Qubit tube (Invitrogen). The concentration of samples was measured using the Qubit tube. The volume required to aliquot 1 ng of the X fragment and Y fragment from the same cell was calculated, and these volumes were combined in a new Lo-bind tube.

### 3.13 Library preparation

The Illumina DNA Prep protocol was followed, using IDT for Illumina DNA/RNA UD Indexes Set A, Tagmentation (96 indexes, 96 samples) (Illumina 20027213). Each single cell should have a unique pair of indexes. The library quality of the cleaned-up library was checked by running 1 µL on a TapeStation D5000 microwell. The libraries were combined and diluted to a starting concentration of 2 nM as per the manufacturer’s instructions.

Stopping point: Samples can be stored at −20°C for 30 days.

### 3.14 Sequencing

Libraries generated using Illumina DNA Prep were compatible with a wide range of Illumina sequencers, including HiSeq, iSeq 100, MiniSeq, NextSeq, and NovaSeq technologies.

The iSeq cartridge and flow cell were prepared as per the manufacturer’s instructions (Illumina 20031371). A 2% PhiX (Illumina FC-110-3001) spike-in was added. The sample sheet loaded onto iSeq was checked to ensure correspondence to the sample sheet from the library preparation. The cartridge was loaded, and the run was performed as per the manufacturer’s instructions. After running, the data were downloaded and backed up on an external hard drive.

### 3.15 Data preprocessing

GitHub repository: https://github.com/alanfoleynibrt/SingleCellmtDNA.

The bioinformatics pipeline is available in the above GitHub repository. Initial processing of data was performed in Linux, and figures were generated in R. All raw FASTQ data analyzed are made available in this pipeline. A step-by-step protocol is also provided.

Briefly, trim_galore (0.4.3) trimmed adapter sequences in FASTQ files. Bowtie-2 (2.3.4.1) mapped reads to the KX576660.1 CHO mtDNA reference genome. Picard (1.199) tools identified duplicates (MarkDuplicates), added read groups (AddOrReplaceReadGroups), and built a BAM index (BuildBamIndex). Gatk3.8-0 realigned indels (IndelRealigner) and recalibrated bases (BaseRecalibrator). Two separate mutation-calling software programs were used: loFreq_star-2.1.2 and VarScan. v2.3.9. When a mutation was called by both, it was selected for analysis. If a mutation allele frequency was between 0.04 and 0.96, it was considered “heteroplasmic.” The potential impact of identified mutations was predicted using snpEff. In tandem, analysis was repeated using a shifted mtDNA reference genome to achieve complete coverage over the D-loop region. Unshifted mutation calls were concatenated with those from the shifted reference sequence to provide full coverage. ggplot2 in R was used to generate figures.

## 4 Results

### 4.1 Sample generation to demonstrate the method

The overall aim of this project was to demonstrate a method to analyze single CHO cells. We first confirmed that sorting 0 cells led to no amplification of mtDNA, and amplification of our single cells led to specific 8.5-kb bands indicative of mtDNA (Figure 2G). To demonstrate the functionality of our workflow as a whole, we sorted four single cells and a bulk sample (4,000 cells) into 5 µL of TCL lysis buffer. Samples were purified, split into two equal aliquots, and separately amplified by LRPCR, which were then visualized on an agarose gel. mtDNA-specific bands were excised for both amplified fragments, as indicated by the red boxes in Figure 2G. Amplicons were recovered by gel purification. After quantifying the dsDNA using the Qubit 1x dsDNA HD Kit, equimolar quantities of each fragment from the same cell were added to a single tube.

For library preparation, the Illumina DNA Prep protocol (20018705) with IDT for Illumina DNA/RNA UD Index Set A was implemented. Each sample was separately assigned unique indices. Separate libraries were combined, diluted to 2nM, and loaded onto Illumina iSeq for PE150 sequencing. A 2% PhiX library control spike-in was added. iSeq had the option to include a “sample sheet” to which the index combinations were added. iSeq was run to completion with an output of fastq.gz files ready for the bioinformatics pipeline.

### 4.2 Bioinformatic analysis

**FIGURE 3:**
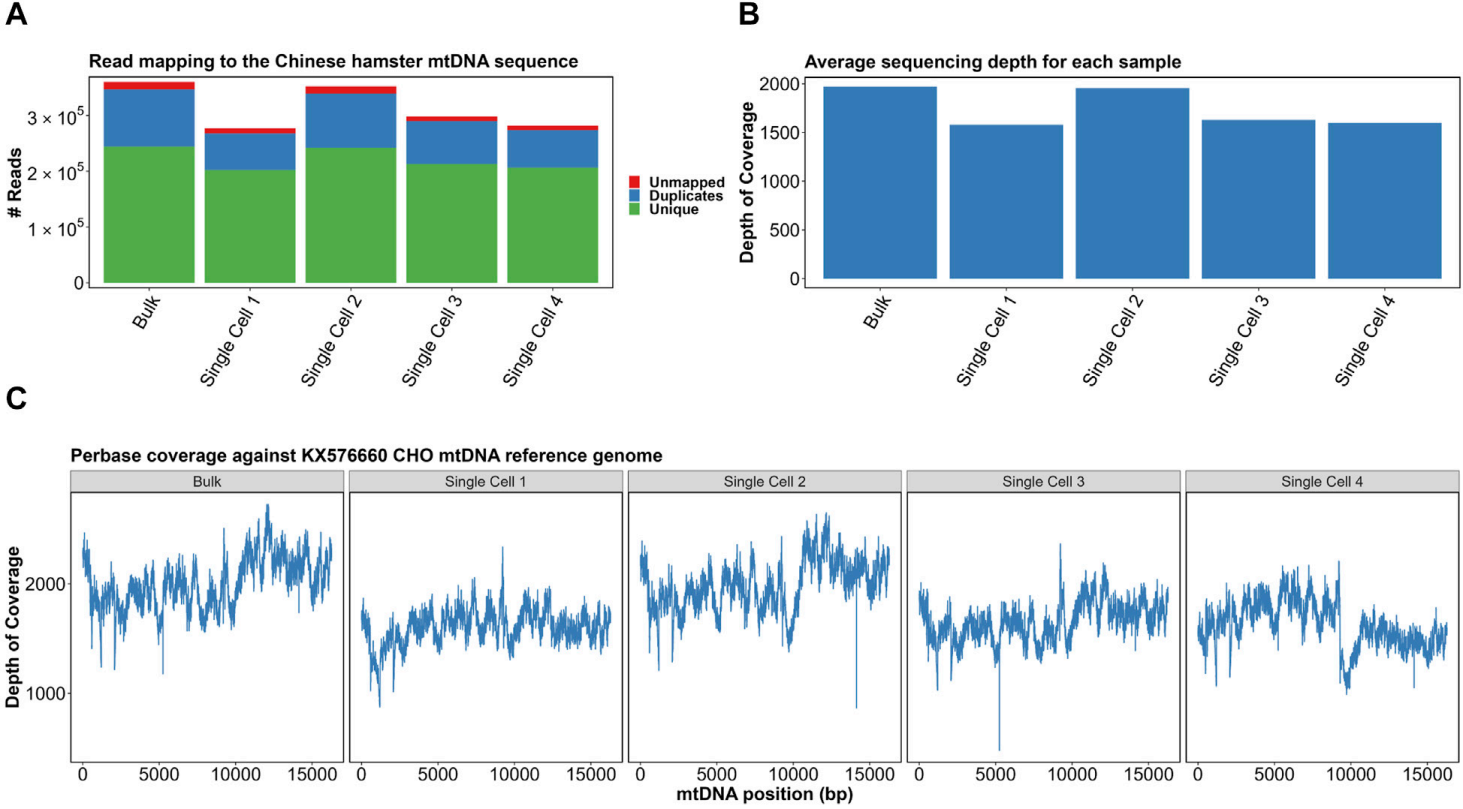
**(A)** Read mapping of samples to the KX576660 CHO mtDNA reference genome. **(B)** Average per-base sequencing depth of each sample. **(C)** Per-base coverage of four single cells and a bulk sample with correction around 0 coordinate. Made with BioRender.

Adapter sequences were removed from the reads, which were then mapped against the CHO KX576660.1 mtDNA reference genome. As was performed previously in the bulk analysis of CHO mtDNA, PCR duplicates were identified (Figure 3A) and removed from the analysis (Kelly et al., 2017). The range of duplicate reads was 23.8%–29.3%, with the highest proportion in the mixed population. Furthermore, indel realignment and base recalibration were used to cater to the effects of indels on read mapping.

After excluding duplicate and unmapped reads, all samples had an average sequencing depth of >1,500×–above 1,000× required for “ultra-deep sequencing” categorization (Figure 3B). Per-base coverage of all samples confirmed complete and even mapping of sequencing reads (Figure 3C). The mapping indicated no strong bias for any particular region. Together, this confirmed our scmtDNAseq protocol had been successful. The great value of single cell sequencing of mtDNA at such a great depth is the ability to analyze with confidence the differences in the sequenced reads when compared to the reference genome, i.e. mutations. Heteroplasmy can be quantified by the proportion of mtDNA copies that contain the mutation. For example, if 50/100 reads contain a mutation, the allele frequency is determined as 0.5.

**FIGURE 4:**
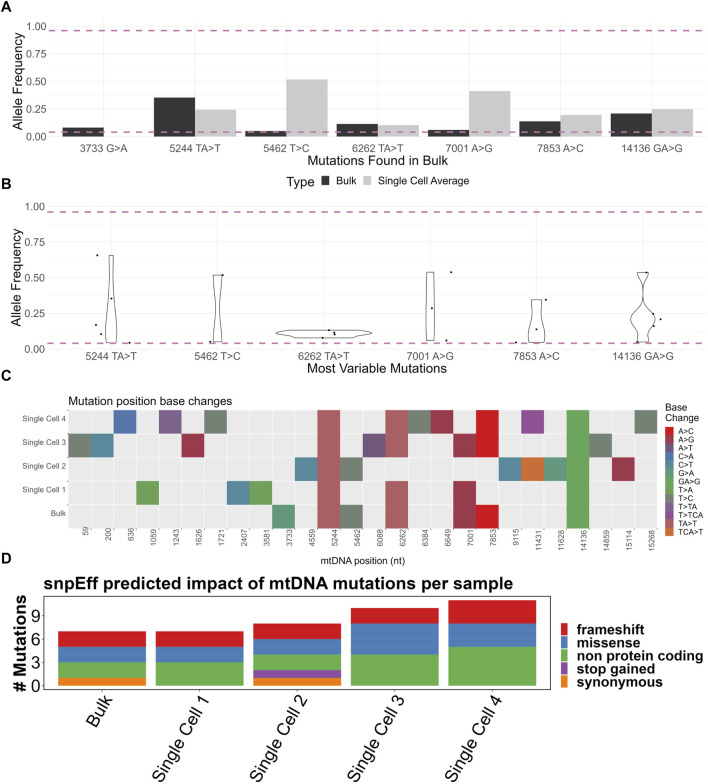
**(A)** Comparison of bulk sample allele frequencies to the average of four single cells’ allele frequencies. The mutation must be heteroplasmic (between 0.04 and 0.96 allele frequency) in the bulk sample. **(B)** Violin plot of “most variable” mutations which must be heteroplasmic in at least two of four single cells. **(C)** Base change heatmap of heteroplasmic mutations (between 0.04 and 0.96 allele frequency) in four single cells and a bulk sample. **(D)** SnpEff-predicted impact of heteroplasmic (between 0.04 and 0.96 allele frequency) mtDNA mutations of four single cells and a bulk sample. Made with BioRender.

**FIGURE 5:**
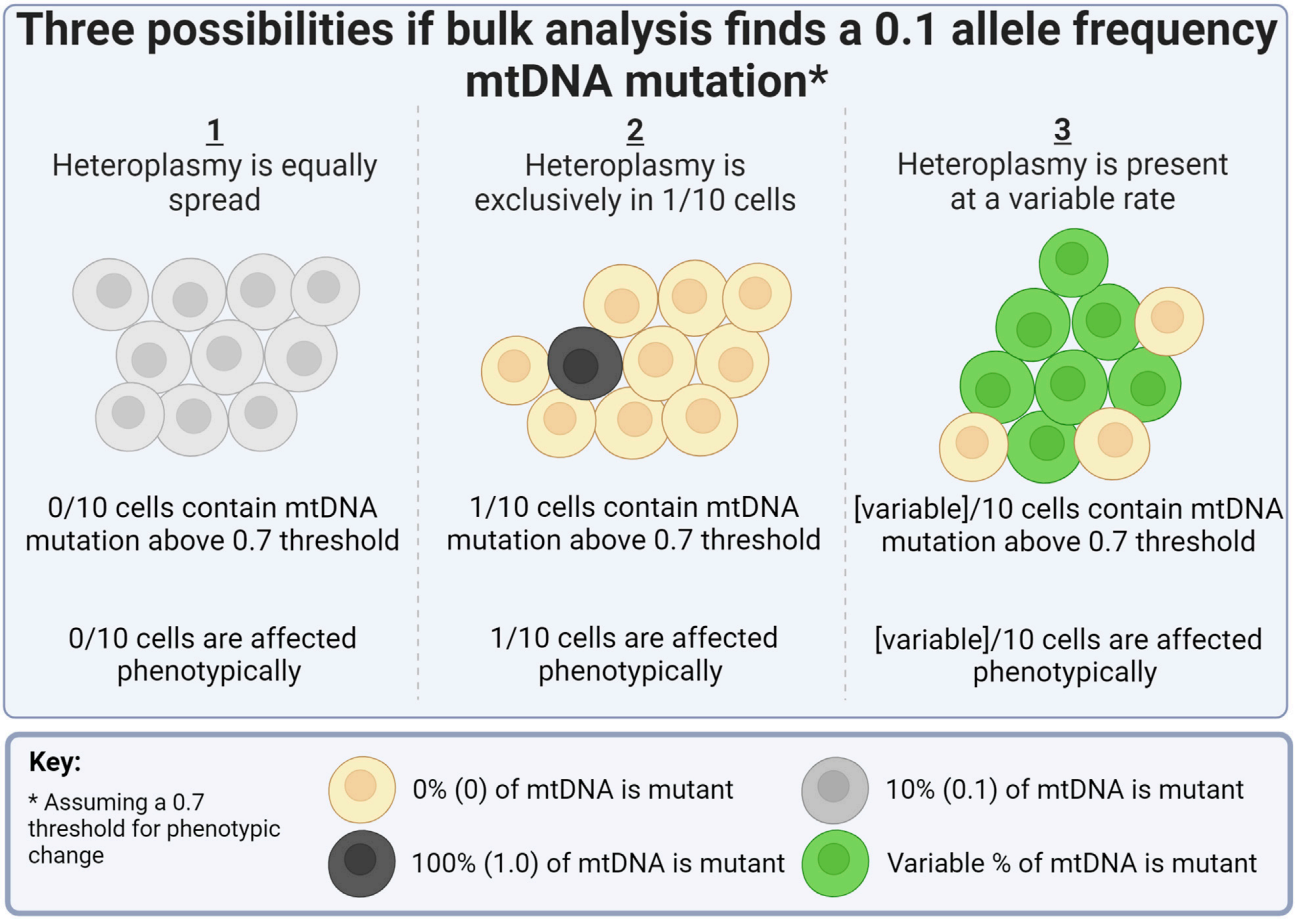
Representation of how the bulk analysis of heteroplasmy can obfuscate single cell orientations. Made with BioRender.

A total of 43 mutations were called among the four samples, of which 17 were indels and 26 SNPs (Supplementary Figure S1B). Of the heteroplasmic mutations in the bulk sample, the single cell average allele frequency varied dramatically from the bulk (Figure 4A). For example, the 5462T>C mutation was 0.05 in the bulk but over 0.5 in the single cell average. This is likely a consequence of the small number of single cell samples sequenced but also suggests that some mutations may exist sporadically at a high frequency in a small number of cells within the population (scenario 2 in Figure 5).

To better assess the variability in the allele frequency among single cells, we developed a list of “most variable” mutations which must be heteroplasmic in at least two out of the four single cells. There was a wide range of allele frequencies among the single cells (Figure 4B). Had only bulk analysis been performed, this range of allele frequency would not have been captured, demonstrating the enhanced resolution possible from single cell analysis.

We next considered whether the mutations among the samples were concordant. We generated a heatmap of mutations from all samples, with each mutation type represented by a color (Figure 4C). The mutations 5244TA>T and 14136T>A were present in all samples. Considering the averaged nature of a bulk sample, one might expect that all the mutations present in the single cells should be equally present in the bulk sample. However, 73% (19/26) of mutation locations were exclusively found in the single cells but not in the bulk sample. One explanation for this is an uneven spread of heteroplasmy among the single cells; in a particular cell, there may be a mutation present above a 0.04 allele frequency, but when averaged across all cells, this allele frequency dips below 0.04. All mutations from the bulk sample were found in at least one single cell, apart from the intragenic 3733G>A. The lack of 3733G>A may be explained by the small number of single cells analyzed.

### 4.3 Predicted phenotypic impact of mutations

Although the presence of mutations is useful for demonstrating intercellular diversity, their phenotypic impact may be limited by many factors, including the mutation’s effect on, for example, the amino acid sequence or tRNA structure. We therefore analyzed mutations based on the predicted impact on the phenotype using the SnpEff software tool (Figure 4D) (Supplementary Figure S1D). Of particular interest, frameshift mutations were observed in protein-coding genes *COX1*, *CYTB*, and *ND4* (Supplementary Figure S1E). At least one frameshift mutation was called in all samples. Only four heteroplasmic mutations were above the 0.5 allele frequency, with most mutations present at low levels. Mapping of mutations against the allele frequency showed the vast majority to be in the 0.04–0.5 range (Supplementary Figure S1F).

## 5 Discussion

### 5.1 Relevance of scmtDNAseq for the biotechnology industry

The heteroplasmic allele frequency is the main determinant of the clinical severity of primary mitochondrial disorders (Nissanka and Moraes, 2020). Previous bulk analysis of CHO mtDNA identified heteroplasmy in clones derived from the same parental host, indicating at least three levels of heterogeneity: 1) production run to production run, 2) cell line to cell line, and 3) clone to clone (Kelly et al., 2017).

However, bulk analysis of heteroplasmy fails to identify the allele frequency differences between individual cells in a population, which is the main determinant of the phenotypic impact (Figure 1A). A heteroplasmic threshold within a single cell must be passed before impairment in OXPHOS and wider phenotypic effects (Rossignol et al., 2003). The exact threshold is highly variable depending on the mutation, cell type, and tissue but generally ranges between 0.6 and 0.9. For example, individuals with a mtDNA mutation in 3243A>G experience diabetes and autism at a low allele frequency of 0.1–0.3, but in individuals with a higher allele frequency, the severity increases with more severe encephalomyopathies at 0.5–0.9 and even perinatal lethality as the allele frequency approaches 1.0 (Picard et al., 2014). However, it is still unclear how the heteroplasmy load among the individual cells affects the overall phenotype. Single cell analysis is therefore critical to reveal the true phenotypic effect of heteroplasmy.

To illustrate this, consider if there was a hypothetical mutation with a threshold of 0.7, above which phenotypic changes would manifest in an individual cell. If bulk analysis identified this critical mutation at a frequency of 0.1 in 1 million cells (Figure 5), three very different conclusions could be arrived at: 1) all cells contain the mutation at a 0.1 allele frequency and are, therefore, all unaffected phenotypically; 2) 10% of cells contain the mutation at a 1.0 allele frequency, and therefore, only 10% of cells are affected phenotypically, or 3) cells contain the mutation at a variable rate (0–1.0), and therefore, the population is affected at a variable rate.

Bearing in mind the strive for homogeneity in drug production, the implications of these scenarios are significant. If a particular heteroplasmy profile affected the product quality, for example, perhaps only a subset of the cells produces the product at a high quality, in which case, the remainder could be identified and potentially excluded to improve bioreactor performance. Equally, perhaps a cell line could be engineered with a favorable heteroplasmy profile to improve the bioreactor performance. Further work is needed to understand the link between mitochondrial heteroplasmy and cellular behavior in recombinant protein production, but single cell analysis should contribute significantly in this regard.

Although the five samples here are demonstrative and not enough for strong statistical conclusions, certain observations were made. The bulk population had the lowest number of reliably detectable mutations (Supplementary Figure S1B). All mutations in the bulk population, bar one, were found in at least one single cell (Figure 4C). This demonstrated the improved resolution of mutation detection using a single cell approach. A great range of allele frequencies in “most variable” mutations was observed (Figure 4B), further indicating an uneven spread of heteroplasmy among the four cells, reminiscent of scenario 3 in Figure 5.

Zambelli et al. previously compared mtDNA sequenced from human fibroblast cells at both bulk and single cell levels (Zambelli et al., 2017). They equally observed an uneven spread of heteroplasmy among single cells. Strikingly, for mutations 12071T>C and 12850A>G, the bulk allele frequency was 0.10 and 0.16, respectively, but completely absent in all but a select few single cells at near 1.0 allele frequency. Could this imply that in an industrial CHO cell culture, there are select individual cells with exceptionally high allele frequency mtDNA mutations with concomitant metabolic impairment?

High-impact mutations observed here in *CYTB* (Supplementary Figure S1D) would change the encoded amino acid sequence. The phenotypic effects of *CYTB* mutations are well-established in human disease, where patients experience highly variable severities of myopathy and muscle weakness (Blakely et al., 2005). *CYTB* mutations in yeast models can cause severe decreases in respiratory function (Fisher et al., 2004). In a bioreactor, mutated *CYTB* single cells (above a phenotypic threshold) may be one of many contributing factors to the heterogeneity observed among clonally derived CHO populations.

### 5.2 Incorporating heteroplasmy for clonal populations

Clonal populations of industrial CHO cell lines start from a single cell to encourage “genetic robustness” (Wurm and Wurm, 2017). This step could also facilitate the future selection of favorable heteroplasmic profiles, which improve the metabolic qualities of the bioreactor run. However, even if a clonal population derives from a CHO cell with a favorable heteroplasmic profile, genetic heterogeneity within clonal populations is an inevitability due to DNA replication errors, made more pertinent for mtDNA, owing to a greater mutation rate than nuclear DNA (Chial, 2008).

Additionally, during mitosis, mitochondria are divided among daughter cells (Mishra and Chan, 2014). The many divisions throughout a standard bioreactor run could result in uneven distributions of heteroplasmy by random chance, which may, therefore, encourage heterogeneous metabolic phenotypes. Heteroplasmic allele frequencies have been observed to dynamically change over a human B-lymphocyte 28-day cell culture (Zhang et al., 2019). The 15153G>A allele frequency increased from 0.50 on day 0 to 0.78 on day 14 and then decreased to 0.31 on day 28. In total, three mutations revealed a very similar increase-to-decrease trend, and their 0.30–0.90 frequency differentials are in a range likely to affect the phenotype. Therefore, future research would benefit from analyzing the change in heteroplasmy over the time period of a typical bioreactor run.

### 5.3 Technical aspects to calling mtDNA mutations

Previously, Zambelli et al. (2017) suggested a heteroplasmy threshold of 0.015 as sufficient when using 30 PCR cycles for mtDNA, in line with previous single cell analyses (He et al., 2010; Zhang et al., 2012). Zambelli et al. also compared results when using 35 cycles instead of 30, only finding differences in very low-allele frequency mutations (>0.015). Upon this background, we considered a greater PCR cycle number of 35, along with a higher allele frequency cutoff of 0.04, to be sufficient. Importantly, we make no claims that our allele frequencies are 100% accurate. Most obviously, we are only sequencing a subset of the total mtDNA copies in each single cell, which could lead to a sampling error. Another possible contribution to the error could be PCR bias. We do not expect a great PCR bias due to the Illumina library preparations as MarkDuplicates should tag fragments with the same origin. For our LRPCR, however, there may be some bias; for this reason, we kept the PCR cycle low at 35 cycles to minimize this bias; however, it is important to note that decreasing the cycle number further could have resulted in a technical limitation because of the small starting mtDNA mass from a single cell.

This also precludes the use of “PCR-free” techniques that may better reflect the “true” allele frequency. Nevertheless, a previous investigation into PCR bias in mtDNA sequencing concluded that PCR-based amplification was suitable for “generating fully accurate mtDNA sequences” and “assessing heteroplasmy for single point mutations with high accuracy” (Legati et al., 2021). Furthermore, they noted a limitation in not “detecting break positions and heteroplasmy of single large deletions.” Therefore, we expect our protocol to be sufficient for heteroplasmic variants to a high accuracy but not large-scale deletions.

Of note, we sequenced single cells selected with a greater AB-FITC stain. This theoretically selects for cells with greater mAb production, which could have imparted bias in the heteroplasmic profiles. Perhaps single cells with greater heteroplasmic burden are more likely to exhibit metabolic impairment. Thus, our selection of greater AB-FITC stain may have concomitantly selected for single cells with lesser metabolic impairment. For research purposes, it may be useful in the future to have an unbiased selection of single cells to fully interrogate the connection of heteroplasmy to metabolic impairment. Conversely, in terms of incorporating a selection step for future clonal CHO populations, it may also be useful to use the AB-FITC signal to preemptively select higher producers.

In conclusion, a reliable method to amplify and analyze mtDNA from single CHO cells was demonstrated (scmtDNAseq). This approach should help better understand the degree and likely impact of heteroplasmy on recombinant protein production in CHO cells.

## Data Availability

The original contributions presented in the study are publicly available. This data can be found here: https://www.ncbi.nlm.nih.gov/bioproject/PRJNA1023156/.

## Supplementary material

SUPPLEMENTARY FIGURE S1
**(A)** Tapestation (4200) image of mtDNA LRPCR for dilutions of a single cell. Negative control is 0 cells, and positive control is 10 cells. Dilutions were made from 1/10 to 1/100,000. **(B)** Number of heteroplasmic mutations (between 0.04 and 0.96 allele frequency) in four single cells and a bulk sample. **(C)** Allele frequency heatmap of heteroplasmic (between 0.04 and 0.96 allele frequency) mutations in four single cells and a bulk sample. **(D)** snpEff-predicted impact of heteroplasmic (between 0.04 and 0.96 allele frequency) mutations in four single cells and a bulk sample. **(E)** Number of mutations per gene in four single cells and a bulk sample. **(F)** Allele frequency distribution of heteroplasmic (between 0.04 and 0.96 allele frequency) mutations in four single cells and a bulk sample. Made with BioRender.
