# Supplementary figures and images for: A complete workflow for single cell mtDNAseq in CHO cells, from cell culture to bioinformatic analysis
Source: Front Bioeng Biotechnol. 2024 Feb 19;12:1304951. doi: 10.3389/fbioe.2024.1304951 (PMC10910102; doi:10.3389/fbioe.2024.1304951)

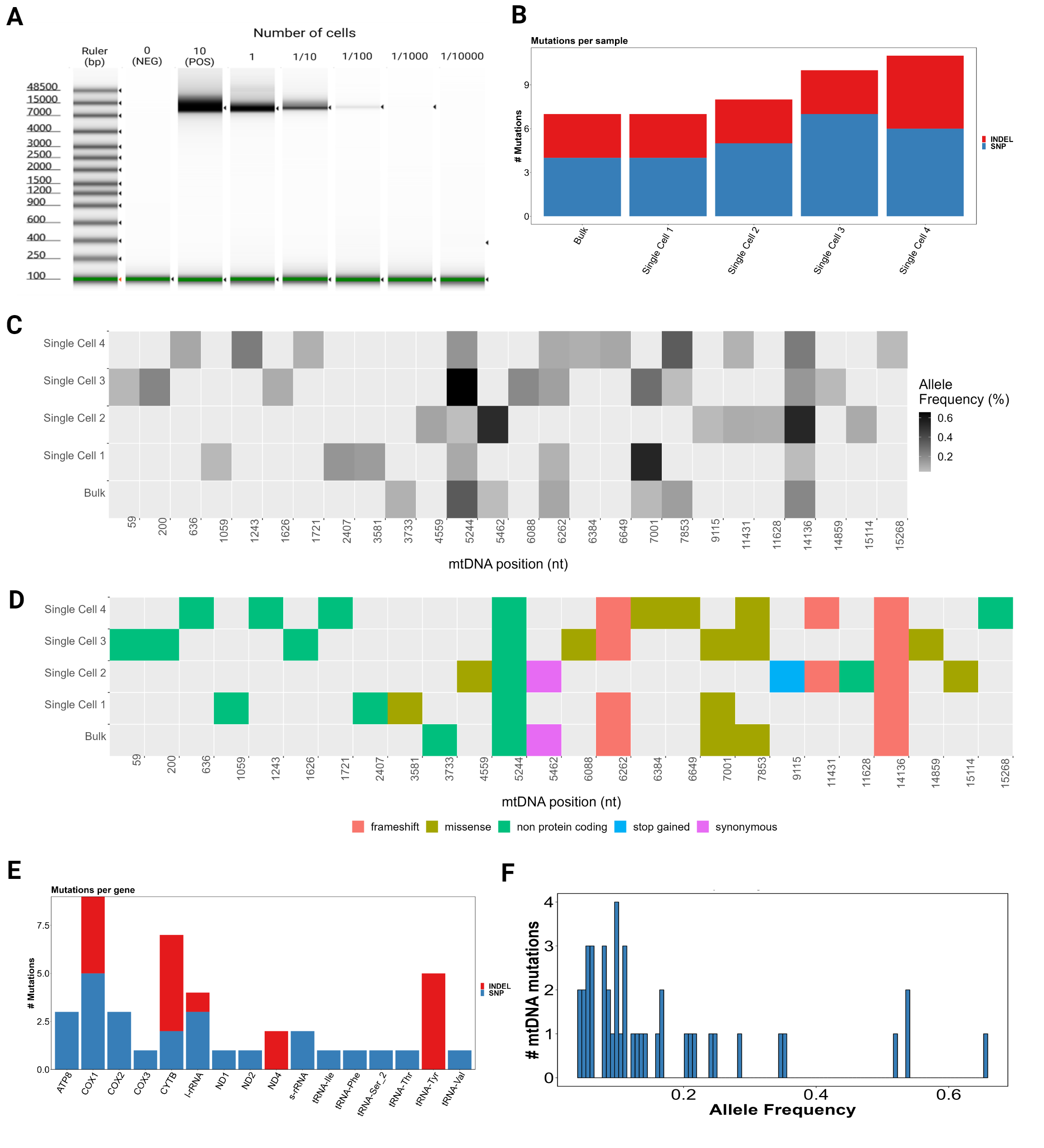

Supplement: Supplementary file 1 [file Image1.png]
